# Evaluation of the Ethanolic Leaf Extract of Abutilon indicum on Isonicotinic Acid Hydrazide-Induced Proinflammatory Marker Gene Expression Changes

**DOI:** 10.7759/cureus.50102

**Published:** 2023-12-07

**Authors:** Mannala Sunil, T Vedavijaya, Rohit Singh Thakur, Karuna Sree P, Venkata Ramana Yella, Suresh Babu Sayana

**Affiliations:** 1 Department of Pharmacology, Meenakshi Academy of Higher Education and Research, Chennai, IND; 2 Department of Pharmacology, Meenakshi Ammal Dental College and Hospital, Chennai, IND; 3 Department of Pharmacology, Malla Reddy Institute of Medical Sciences, Hyderabad, IND; 4 Department of Pharmacology, All India Institute of Medical Sciences, Kalyani, IND; 5 Department of Pharmacology, Government Medical College and General Hospital, Suryapet, IND

**Keywords:** proinflammatory tnf-α inflammation pathway, nf-κb signaling, silymarin, abutilon indicum, inh

## Abstract

Background: Abutilon indicum, widely found in India, Sri Lanka, and parts of America and Malaysia, is renowned for its rich bioactive compounds including alkaloids, flavonoids, and sesquiterpene lactones. Due to its diverse pharmacological potential, it has garnered significant attention in traditional medicine. In particular, the ethanolic leaf extract of Abutilon indicum (ELEAI) has demonstrated anti-inflammatory effects, notably targeting the 5-lipoxygenase enzyme pivotal in inflammatory responses.

Objective: This study aimed to elucidate the impact of the ELEAI on proinflammatory marker gene expression induced by isoniazid (INH).

Methods: A total of 36 rats were systematically divided into six experimental groups. The control group received DMSO orally for the initial 30 days followed by distilled water for the subsequent 30 days. The INH group received a daily dose of INH (30 mg/kg b.w., i.p.) for 30 days and the rats were then sacrificed on day 30. The ELEAI (250 mg/kg) group was administered INH daily for 30 days, followed by daily post-treatment with ELEAI (250 mg/kg) for another 30 days. Similarly, the ELEAI (500 mg/kg) group received INH daily for 30 days, followed by daily post-treatment with ELEAI (500 mg/kg) for another 30 days. The silymarin (SIL) group was given INH daily for 30 days, followed by post-treatment with SIL at a dose of 100 mg/kg body weight daily for the subsequent 30 days. Finally, the ELEAI (500 mg/kg) alone group was administered distilled water orally for the first 30 days and then received ELEAI at a dose of 500 mg/kg b.w. orally once daily for the next 30 days.

Results: Continuous INH exposure for a month led to a pronounced increase in proinflammatory genes like TNF-α, TGF-β, and NF-kB and a decrease in the IkB gene in rat liver tissues. Subsequent treatment with SIL (100 mg/kg) and ELEAI (250 and 500 mg/kg) post-INH exposure resulted in a marked decrease in proinflammatory genes and a surge in IkB expression.

Conclusion: The findings suggest that the ELEAI exerts a dose-responsive influence on proinflammatory activities. Notably, A. indicum counteracts inflammation, especially that triggered by bradykinin and prostaglandins. The ELEAI showcases promising therapeutic potential, exhibiting both pro and anti-inflammatory properties and antiproliferative characteristics.

## Introduction

Drug-induced liver complications present a significant challenge in hepatology due to the extensive variety of medications used in healthcare, some of which can cause acute liver failure when consumed in excess [1,2]. This includes several herbs and dietary supplements known for their hepatotoxic effects. Drug-induced liver injury (DILI) can be intrinsic, where effects are dose-dependent and anticipated within a short duration, or idiosyncratic, which are usually unpredictable and manifest after a prolonged period [3,4]. Various elements such as drug pharmacokinetics, interactions with immune modulators, and the body's atypical immune response to certain drugs or their metabolites can influence susceptibility to idiosyncratic DILI [5].

Numerous medications are suspected of inducing liver harm, with some like dapsone, acetaminophen, and specific anti-tuberculosis drugs, including isoniazid (INH), having a verified record. Tuberculosis, a significant concern in various regions, often employs INH for treatment, notwithstanding its potential hepatotoxic side effects. INH's metabolism in the liver involves several intermediate compounds that can cause liver cell damage [6-8]. Notably, INH's metabolism can also lead to the generation of reactive oxygen species (ROS), further implicating its hepatotoxic nature [9-14].

Flora plays a pivotal role in ethnomedicine, with an impressive portion of higher plants having medicinal applications. While primary plant metabolites encompass basic organic compounds, secondary metabolites derived from them possess diverse biological activities, serving as foundations for various drug developments. One such plant, Abutilon indicum, native to the warmer regions of India, has traditional therapeutic applications for numerous ailments [1,10,11]. Its phytochemicals have shown potential hepatoprotective properties in preliminary studies, validating its traditional use [12].

Proinflammatory markers like tumor necrosis factor-alpha (TNF-α) play multi-faceted roles in liver health [13]. The varying roles of TNF in liver pathology underscore the need for targeted therapies. Furthermore, increased expressions of TNF-α, TGF-β, and NFΚB are indicators of liver damage from various hepatotoxic drugs. Addressing the inflammation induced by drugs like INH, especially in the context of these markers, is crucial.

## Materials and methods

Methodology

Chemicals and Reagents

INH was sourced from Sigma Chemicals, Hyderabad, India. Abutilon indicum plant samples were harvested from various locations in Telangana and later confirmed for authenticity by an expert from the Botany Department, Osmania University, Telangana, India. All other chemicals required for this research were procured from Sigma-Aldrich Company.

Preparation of the Ethanolic Extract

Abutilon indicum leaves were washed with sterilized distilled water, dried, and ground into a fine powder. This powder was subjected to extraction using a 70:30 ethanol-water mixture in a Soxhlet extractor for three days, maintaining a temperature of 60°C. The liquid extract was then concentrated by removing the solvent on a rotary evaporator, ensuring the temperature did not exceed 50°C. This resulted in a light brown solid extract, which was reconstituted in sterile distilled water and stored in a cool, dark place until further experiments.

Male Wistar albino rats, weighing between 150 and 200 g, were employed for this study. These animals were kept in sanitary polypropylene cages with a consistent laboratory environment - a temperature of about 22°C and a 12-hour light-dark cycle. The rats had unrestricted access to standard feed and water.

Experimental Protocol

The 36 rats were systematically segregated into six groups: Group I: Served as a control, given DMSO 1% orally for the initial 30 days followed by distilled water for the subsequent 30 days, Group II: Received a daily dose of INH (30 mg/kg b.w..i.p.) for 30 days and were then sacrificed on day 30, Groups III and IV: Administered INH daily for 30 days, followed by a daily post-treatment with ethanolic leaves extract of Abutilon indicum (ELEAI) at concentrations of 250 mg/kg and 500 mg/kg body weight, respectively, for another 30 days, Group V: Given INH daily for 30 days and then post-treated with SIL at 100 mg/kg body weight daily for the subsequent 30 days, and Group VI: Administered distilled water p.o. for first 30 days and ELEAI dose of (500 mg/kg b.w . po) once daily for next 30 days.

On the 60th day, all rat groups, except Group II (which was sacrificed on the 30th day), were euthanized. INH was prepared in dimethyl sulfoxide, while the ELEAI was reconstituted in distilled water. Liver tissues were processed to isolate total RNA using the TRIzol reagent as per the instructions provided by Invitrogen. The quality and amount of extracted RNA were determined using a NANODROP200 spectrophotometer (Thermo Fisher Scientific, Waltham, MA, USA). Specific primers for TNF-α, transforming growth factor-beta (TGF-β), nuclear factor kappa B (NF-κB), and IκB (NF-κB inhibitor) were synthesized by Sigma Genosys, Bangalore, India. The reverse transcription and amplification processes were performed, and the resultant products were visualized on an agarose gel. Amplified genes' intensity was analyzed using a Kodak Digital Science system (Eastman Kodak Company, Rochester, NY), and expression levels were normalized against β-actin mRNA levels for each sample.

All animal experiments adhered to guidelines and were approved by the Institutional Animal Ethics Committee of Malla Reddy Institute of Medical Sciences, Hyderabad, India (Approval No: MRIMS-IAEC-CCSEA[02/2023]). All data were securely stored using MS Excel 2019 (Microsoft Inc, Redmond, Washington). For each variable, descriptive statistics were calculated, including count, frequency, and mean ± standard deviation (SD). One-way ANOVA was utilized to compare the means across various dosages or drugs, with a significance level set at p ≤ 0.05.

## Results

The results demonstrate the significant impact of the ELEAI on INH-induced proinflammatory marker gene expression, with SIL (silymarin) used as a standard for comparison. In the control group, baseline levels of proinflammatory markers were observed, characterized by low TNF-alpha, TGF-β (transforming growth factor-beta, and NFk-β (nuclear factor-kappa β) expression but high IkB expression. In contrast, the INH group exhibited elevated levels of TNF-alpha, TGF-B, and NFkB, indicating a pro-inflammatory response to INH treatment, although these changes were not statistically significant. Importantly, when ELEAI was introduced at concentrations of 250 and 500, it led to a noteworthy reduction in TNF-alpha and NFkB expression, which was statistically significant, suggesting a potent anti-inflammatory effect. Additionally, IkB expression increased significantly with ELEAI treatment, indicating its role in inhibiting NFkB activation. TGF-B expression showed variable changes, suggesting a more complex interplay. These findings underscore the potential of ELEAI in alleviating INH-induced inflammation and its promising anti-inflammatory properties.

Furthermore, the results from the INH+SIL (Silymarin) group, where silymarin served as a standard, also demonstrated a significant reduction in TNF-alpha and NFkB expression, along with an increase in IkB expression, reinforcing the anti-inflammatory potential of ELEAI. The ELEAI 500 group, without INH treatment, displayed low baseline levels of proinflammatory markers, indicating the safety and potential anti-inflammatory properties of ELEAI even in the absence of INH-induced stress. These results collectively highlight the anti-inflammatory properties of ethanolic leaf extract of Abutilon indicum and suggest its potential therapeutic utility, particularly in managing inflammation associated with conditions like INH treatment (Table 1).

**Table 1:** Effect of Ethanolic Leaf Extract of Abutilon Indicum on INH-Induced Proinflammatory Marker Gene Expression Changes INH-Isonicotinic acid hydrazide; ELEAI- ethanolic leaf extract of Abutilon indicum; TGF-β: transforming growth factor-beta; TNF-α: tumor necrosis factor-alpha; NF-κB: nuclear factor-kappa B; IκB: inhibitor of kappa B

Group	TNF-alpha	TGF-B	NFkB	IkB	P-value
Control	0.5 ± 0.06	0.4 ± 0.04	0.5 ± 0.03	2.1 ± 0.13	<0.001
INH	1.1 ± 0.09	1.1 ± 0.09	1.1 ± 0.13	0.9 ± 0.02	0.08
INH+ELEAI 250	0.8 ± 0.05	1.2 ± 0.08	0.8 ± 0.04	1.2 ± 0.13	<0.001
INH+ELEAI 500	0.6 ± 0.05	1.1 ± 0.15	0.6 ± 0.03	1.1 ± 0.06	<0.001
INH+SIL	0.7 ± 0.04	1.1 ± 0.12	0.6 ± 0.03	1.3 ± 0.08	<0.001
ELEAI 500	0.60 ± 0.09	0.4 ± 0.03	0.5 ± 0.03	2.14 ± 0.14	<0.001

In this study, gene expression analysis was conducted on liver tissues to investigate the impact of the experimental conditions on the expression of specific genes related to inflammation. The RNA isolation process utilized TRIzol reagent according to the Invitrogen instructions, ensuring the extraction of high-quality RNA. To assess the quality and quantity of the isolated RNA, a NANODROP200 spectrophotometer was employed.

For the analysis of gene expression, specific primers targeting key genes such as TNF-α, transforming growth factor-beta (TGF-β, nuclear factor kappa B (NF-κB), and IκB (NF-κB inhibitor) were custom synthesized by Sigma Genosys in Bangalore, India. Subsequently, the reverse transcription and amplification processes were carried out, and the resulting amplified gene products were visualized on an agarose gel. The intensity of these amplified genes was quantitatively assessed using a Kodak Digital Science system.

**Figure 1:**
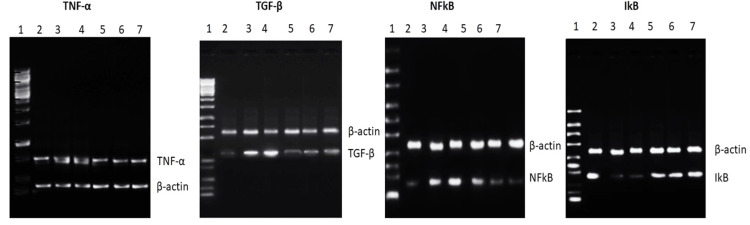
Analysis of Inflammatory Gene Expression in Liver Tissues: Experimental Conditions and Molecular Insights Effect of ELEAI on INH-induced changes in the gene expression of tumor necrosis factor alpha (TNF-α), transforming growth factor beta (TGF-β), nuclear factor kappa B (NF-κB), IκB (inhibitor of NF-κB) in liver tissue of rats. A. Qualitative expression of the above genes. 1 - Marker, 2 - Control, 3 – INH, 4- INH + ELEAI 250 mg/kg, 5 – INH + ELEAI 500 mg/kg, 6 - INH+SIL mg/kg, 7- ELEAI 500 mg/kg.

**Figure 2:**
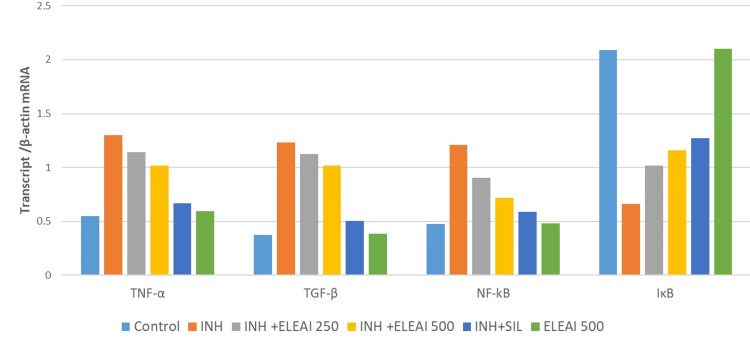
Effect of Ethanolic Leaf Extract of Abutilon indicum on INH-Induced Proinflammatory Marker Gene Expression Changes

To facilitate meaningful comparisons, the expression levels of the target genes were normalized against the expression of β-actin mRNA in each sample. This normalization step ensures that any variations in gene expression observed can be attributed to the experimental conditions and not differences in sample loading or RNA quantity (Figures 1, 2).

## Discussion

Historically, the treatment of tuberculosis (TB) posed significant challenges due to the high mortality rate and low cure rate associated with anti-TB drug regimens. Clinical observations indicated a cure rate of only 11.42%, with a strikingly high mortality rate of 40.9% among patients undergoing anti-TB drug treatment [15-17]. This highlighted the urgent need for interventions to improve treatment outcomes.

One intriguing development in TB treatment was the co-administration of INH, a key anti-TB drug, with plant compounds such as silymarin and curcumin. This combination therapy led to a remarkable transformation in treatment outcomes. The cure rate substantially increased to 41.3%, and the mortality rate plummeted to a mere 3.8% [17]. These findings underscored the potential of plant-based compounds to enhance the effectiveness of anti-TB drugs and reduce the risk of treatment-related mortality.

INH-related hepatic toxicity is a well-documented concern in TB treatment. Notably, this toxicity often manifests without noticeable symptoms, making it crucial to rely on serum markers such as aspartate aminotransferase (AST), alanine aminotransferase (ALT), and alkaline phosphatase (ALP) to assess hepatocyte damage [9,15]. Elevations in these liver enzymes signal hepatocellular injury and are indicative of liver dysfunction.

The process of INH metabolism in the liver, primarily catalyzed by the enzyme CYP2E1, results in an increased production of intracellular free radicals, particularly ROS. This surge in ROS leads to oxidative stress within hepatocytes, contributing significantly to INH-induced liver damage [8]. Oxidative stress, in turn, triggers lipid peroxidation and compromises membrane integrity, playing a pivotal role in liver injury.

Several plant extracts and their phytochemical derivatives have emerged as promising candidates for mitigating hepatic damage induced by anti-TB drugs [11]. These natural compounds are endowed with a rich array of phytochemicals that contribute to their hepatoprotective properties [16]. Notably, these compounds play a crucial role in stabilizing hepatocellular membranes, a key mechanism in protecting liver cells from damage [17]. By restoring membrane stability, these plant-derived compounds facilitate the recovery of liver function following drug-induced injury.

Furthermore, INH metabolites have been found to induce lipid peroxidation, a process integral to liver cell damage [18]. The ability of plant-based compounds to counteract this lipid peroxidation and protect against hepatocyte injury underscores their potential as adjunct therapies in TB treatment. In the context of inflammation, tumor necrosis factor-alpha (TNF-α) plays a crucial role as a pro-inflammatory mediator produced by macrophages [19]. It is central to both macrophage function and the initiation of inflammatory responses. Elevated levels of TNF-α can trigger inflammatory pathways, particularly those mediated by nuclear factor-kappa B (NF-κB) [19]. NF-κB, a key player in pro-inflammatory signaling, has been implicated in the onset and progression of INH-induced liver damage.

To regulate inflammation, an inhibitory protein known as IκB typically binds to and neutralizes NF-κB. However, upon stimulation, IκB undergoes phosphorylation, leading to the release of NF-κB from its inhibitory complex [20]. This released NF-κB subsequently translocates to the nucleus, where it promotes the release of various inflammatory agents, including TNF-α [20]. This proinflammatory cascade contributes significantly to liver inflammation and damage associated with INH treatment.

Another crucial player in this context is transforming growth factor-beta (TGF-β), which is involved in cell damage, oxidative stress, and liver fibrosis [21]. Elevated expression of proinflammatory markers such as TNF-α, TGF-β, and NF-κB is a common feature observed in cases of drug-induced liver damage [21]. These findings underscore the intricate interplay of inflammatory mediators in the pathogenesis of liver injury associated with INH treatment.

Limitations

One limitation of this study is the focus on rat models, which may not fully represent the complexities of human physiology and drug responses. Additionally, the study primarily investigates the impact of Abutilon indicum on proinflammatory markers induced by INH without exploring potential interactions with other medications or considering variations in individual responses. Further clinical studies are needed to validate these findings in human subjects and assess potential side effects or contraindications.

## Conclusions

This study demonstrates the potential of the ELEAI as an effective anti-inflammatory agent, countering proinflammatory responses induced by INH in rat liver tissues. The ELEAI exhibits promising therapeutic properties, including antioxidant and anti-inflammatory effects. However, further research is required to assess its clinical applicability and safety in humans. This investigation underscores the valuable role of Abutilon indicum in traditional medicine and its potential as a natural remedy for hepatic conditions.
